# Mitochondria Transcription Factor A: A Putative Target for the Effect of Melatonin on U87MG Malignant Glioma Cell Line

**DOI:** 10.3390/molecules23051129

**Published:** 2018-05-09

**Authors:** Daiane G. Franco, Isabele F. Moretti, Suely K. N. Marie

**Affiliations:** Faculdade de Medicina FMUSP, Universidade de Sao Paulo, Sao Paulo, SP 01246903, Brazil; isabelemoretti@gmail.com (I.F.M.); sknmarie@usp.br (S.K.N.M.)

**Keywords:** melatonin, mitochondria, TFAM, cancer, glioblastoma

## Abstract

The disruption of mitochondrial activity has been associated with cancer development because it contributes to regulating apoptosis and is the main source of reactive oxygen species (ROS) production. Mitochondrial transcription factor A (TFAM) is a protein that maintains mitochondrial DNA (mtDNA) integrity, and alterations in its expression are associated with mitochondrial damage and cancer development. In addition, studies have shown that mitochondria are a known target of melatonin, the pineal gland hormone that plays an important anti-tumorigenic role. Thus, we hypothesized that melatonin decreases the expression of TFAM (RNA and protein) in the human glioblastoma cell line U87MG, which disrupts mtDNA expression and results in cell death due to increased ROS production and mitochondrial damage. Our results confirm the hypothesis, and also show that melatonin reduced the expression of other mitochondrial transcription factors mRNA (TFB1M and TFB2M) and interfered with mtDNA transcription. Moreover, melatonin delayed cell cycle progression and potentiated the reduction of cell survival due to treatment with the chemotherapeutic agent temozolomide. In conclusion, elucidating the effect of melatonin on TFAM expression should help to understand the signaling pathways involved in glioblastoma progression, and melatonin could be potentially applied in the treatment of this type of brain tumor.

## 1. Introduction

Mitochondria are double-membrane organelles that contain their own genetic material, mitochondrial DNA (mtDNA), which is circular, high-copy-number DNA [1,2]. In addition to being an energy source for the cell that produces adenosine triphosphate (ATP) via oxidative phosphorylation, mitochondria are important in other processes, such as calcium homeostasis, fatty acid oxidation, the synthesis of reactive oxygen species (ROS), apoptosis, the cell cycle, and proliferation. Accordingly, the disruption of mitochondrial activity is associated with several diseases, including cancer [3,4,5]. Moreover, mtDNA associates with the internal membrane of mitochondria via a nucleoprotein complex called nucleoid [6,7], and the most abundant protein component of this structure is mitochondrial transcription factor A (TFAM), which belongs to the high mobility group (HMG) protein family [7,8,9]. Although TFAM can bind to mtDNA in a nonspecific manner to promote the packaging and maintenance of genetic material, TFAM also binds to the promoter region to regulate transcription, together with two other transcription factors (TFAB1M and TFB2M) that form a complex with mitochondrial RNA polymerase (POLRMT) [10]. In addition, TFAM also regulates the replication of mtDNA by controlling its copy number [6,11].

TFAM has been considered a potential target for cancer therapy since changes in its expression have been detected in several types of cancer [12,13,14,15]. In glioma, the TFAM RNA and protein levels are upregulated, compared to non-neoplastic brain tissue [12,13]. Nevertheless, the protein levels of TFAM positively correlated with the malignancy of glioma [15], higher RNA levels of TFAM correlated with a better prognosis among patients with grade IV glioma (glioblastoma, GBM) [13].

Glioblastoma, or grade IV astrocytoma, as classified by the World Health Organization (WHO), is a tumor of glial origin restricted to the central nervous system (CNS) that is highly invasive to the surrounding cerebral parenchyma [16,17]. Despite advances in knowledge regarding the molecular biology of astrocytomas that have improved diagnosis and treatment, the prognosis of this condition remains poor, with a maximum survival of 24 months [16,17,18]. The treatment of this cancer currently involves a combination of surgery, radiotherapy, and chemotherapy with temozolomide (TMZ) [16,19,20,21,22].

Melatonin (*N*-acetyl-5-methoxytryptamine), a hormone synthesized from serotonin, acts on several cellular processes, including proliferation, differentiation, invasion, and apoptosis, to result in different effects on non-tumor cells and tumor cells [23,24]. In normal cells, melatonin increases viability and acts as an antioxidant, whereas it activates apoptosis and increases the cellular content of reactive oxygen species (ROS) in tumor cells, effects that depend on mitochondrial activity [25,26]. The use of melatonin as an adjuvant cancer chemotherapy has shown promising results in relation to both improving the efficacy of treatment and reducing the incidence of side effects. The effects of melatonin on mitochondria have been widely explored, however little is known about the effects of melatonin on mtDNA and TFAM expression. We hypothesized that melatonin can reduce the expression of mitochondrial transcription factors (TFAM, TFMB1M, and TFB2M) to decrease the viability of cancer cells due to an imbalance in mitochondrial activity. Using a GBM cell line (U87MG), we showed that melatonin decreased the level of mitochondrial transcription factors, induced mitochondrial membrane depolarization to cause apoptosis, increased intracellular oxidative stress, and delayed the cell cycle. When used as an adjuvant therapy with TMZ, melatonin enhanced the efficacy of chemotherapy by further decreasing cell viability/proliferation.

## 2. Results

### 2.1. Melatonin Decreased the Expression of TFAM, TFB1M, and TFB2M

**Figure 1:**
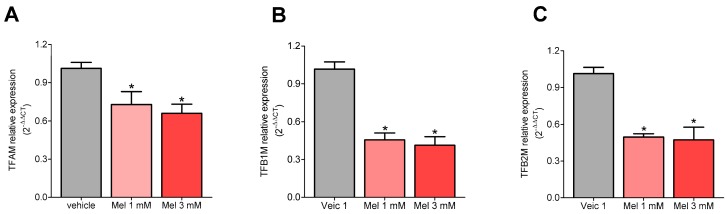
Melatonin inhibits the expression of mitochondrial transcription factor A (TFAM), TFB1M, and TFB2M—Cultured U87MG cells were incubated with melatonin (1 mM or 3 mM) for 72 h, and the medium was exchanged every 24 h. The relative mRNA expression levels of each gene were quantified by qRT-PCR using the geometric mean of the following normalizing genes: Hypoxanthime phosphoribosyl transferase (HPRT), glucuronidase-beta (GUS-B), and TATA-Box binding protein (TBP) [27]. The data are expressed as the relative quantification (2^−ΔΔCt^) compared to the vehicle-treated groups (ethanol 0.3% or 0.9%). Gene expression did not differ in cells treated with vehicle or 0.3% and 0.9% ethanol, and these groups were represented as a single group. From left to right are presented the results for TFAM (**A**), TFB1M (**B**), and TFB2M (**C**). * *p* < 0.05, tested with an analysis of variance followed by the Bonferroni post-hoc correction using GraphPad Prism^®^ version 5, comparing the effect of melatonin to the vehicle group.

We first investigated the ability of melatonin to change the gene expression of transcription factors that act on mitochondria, and the effect of this change on the proper functioning of the organelle and, consequently, the cell. Incubation with melatonin (1 mM or 3 mM) for 72 h reduced the expression of the transcriptions factors *TFAM* (Vehicle: 1.01 ± 0.05%; Mel 1 mM: 0.73 ± 0.10%; Mel 3 mM: 0.66 ± 0.07%), *TFB1M* (Vehicle: 1.04 ± 0.06%; Mel 1 mM: 0.46 ± 0.05%; Mel 3 mM: 0.41 ± 0.07%), and *TFB2M* (Vehicle: 1.02 ± 0.05%; Mel 1 mM: 0.50 ± 0.03%; Mel 3 mM: 0.47 ± 0.10%), compared to the vehicle control group (Figure 1A–C).

### 2.2. Melatonin Decreased the Content of TFAM Protein

**Figure 2:**
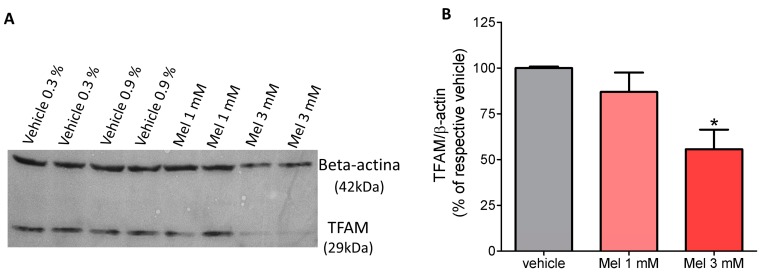
Melatonin decreases TFAM content in U87 GBM cell lineage—(**A**) Representative Western blot image shows the effects of melatonin treatment (1 mM and 3 mM) and their respective vehicle groups (ethanol 0.3% and 0.9%) on the protein TFAM expression. (**B**) The bar graph shows quantitative signal intensities of the protein TFAM expression after normalization with β-actina. TFAM protein cell content did not differ in cells treated with vehicles and these groups were represented as a single bar. * *p* > 0.05 compared to vehicle. The statistical analysis consisted of an ANOVA followed by Bonferroni’s post-hoc test.

Western blotting analysis showed that expression of TFAM at the protein level was decreased following melatonin (3 mM) treatment compared to the vehicle group (ethanol 0.9%), but for the 1 mM concentration, the melatonin effect was variable and the result was not statistically significant. (Figure 2 and Supplementary material—Figure S1, Table S2).

### 2.3. Melatonin Decreased the Transcription of mtDNA but Did Not Affect Replication

**Figure 3:**
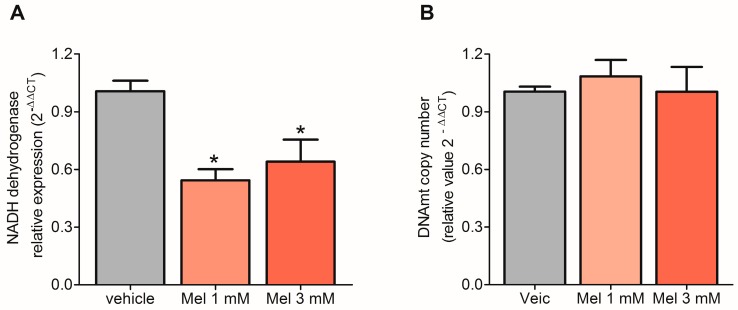
Melatonin inhibits mitochondrial NADH dehydrogenase 1 gene expression but does not affect mitochondrial DNA (mtDNA) replication—Cultured U87MG cells were incubated with melatonin (1 mM or 3 mM) for 72 h, and the medium was exchanged every 24 h. The relative expression of the NADH dehydrogenase 1 gene (**A**) and mtDNA copy number (**B**) were determined by qRT-PCR, using mitochondrial RNA and DNA as a template, respectively. The data are expressed as the relative quantification (2^−ΔΔCt^) compared to the vehicle-treated groups (ethanol 0.3% or 0.9%). Gene expression did not differ in cells treated with vehicle or 0.3% and 0.9% ethanol, and these groups were represented as a single bar * *p* < 0.05, tested with an analysis of variance followed by the Bonferroni post-hoc correction using GraphPad Prism^®^ version 5, comparing the effect of melatonin to the vehicle group.

Since the transcription factors TFAM, TFB1M, and TFB2M are directly related to the regulation of transcription and mtDNA replication, we evaluated the expression of the MT-ND1 gene to ascertain if the effect of melatonin on transcription factors was reflected in mitochondrial gene expression and mtDNA copy number. To examine mitochondrial gene expression and mtDNA copy number, we used the aforementioned primer for the NADH dehydrogenase 1 gene and mRNA and DNA extracted from U87MG cells treated with 1 mM or 3 mM of melatonin for 72 h, respectively. Melatonin reduced the expression of the mtDNA gene MT-ND1 (Vehicle: 1.01 ± 0.05%; Mel 1 mM: 0.54 ± 0.06%; Mel 3 mM: 0.62 ± 0.12%) (Figure 3A), but despite the reduction in TFAM, TFB1M, and TFB2M expression, mtDNA replication appeared unchanged, since the number of copies of mitochondrial genetic material remained the same after treatment with melatonin (Figure 3B).

### 2.4. Melatonin Induced ROS Production

**Figure 4:**
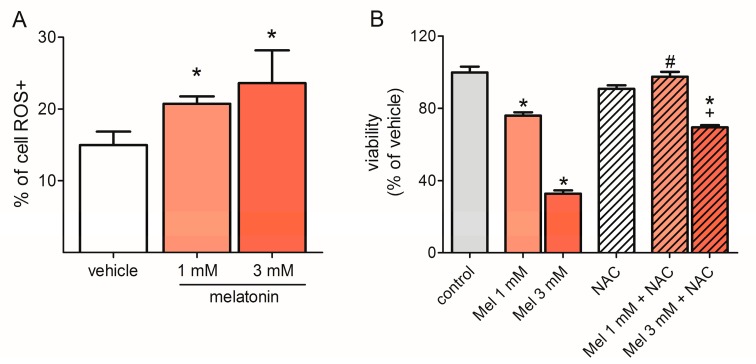
Melatonin increases reactive oxygen species (ROS) production—(**A**) U87MG cells were incubated with melatonin (1 mM or 3 mM) for 72 h, and the medium was exchanged every 24 h. ROS production was assessed by cytometry using the Muse^®^Cell Oxidative Stress kit. The results are presented as the percentage of cells positively labeled for superoxide radicals. ROS production and cell viability did not differ in cells treated with vehicle or 0.3% and 0.9% ethanol, and these groups are consequently represented as a single bar. (**B**) U87MG cell were incubated with vehicle melatonin (1 mM and 3 mM) and *N*-acetyl-l-cysteine (NAC, 10 mM) for 72 h and the medium was exchanged every 24 h. Proliferation was assessed based on the reaction with PrestoBlue (Thermo Fisher Scientific), and the fluorescence was read on a GloMax^®^ 96 Microplate Luminometer (Promega Corporation). The results were presented as a percentage of the control group. As the proliferation did not differ between the vehicle-treated groups, they were represented as a single bar. * *p* < 0.05 compared to vehicle/control, # *p* < 0.05 compared to the group treated with 1 mM melatonin, and + *p* < 0.05 compared to the group treated with 3 mM melatonin. The statistical analysis consisted of an ANOVA followed by Bonferroni’s post-hoc test.

To verify that melatonin increases oxidative stress in U87MG cells, we evaluated the production of superoxides as an indicator of ROS production using cytometry, based on the reaction of total cellular superoxide with dihydroethidium (DHE). The result showed that melatonin increased ROS production to 20.73 ± 1.03% at a concentration of 1 mM, and 23.62 ± 4.56% at a concentration of 3 mM, compared to the vehicle group (14.97 ± 1.89%, Figure 4A). To verify if the increase of ROS induced by melatonin has an effect on cell viability, a known ROS scavenger, *N*-acetyl-l-cysteine (NAC, 10 mM), was used. Figure 4B shows that the antioxidant agent reverts the 1 mM melatonin-induced viability reduction and about 40% the effect of melatonin 3 mM.

### 2.5. Melatonin Induced Mitochondria Depolarization and Apoptosis

**Figure 5:**
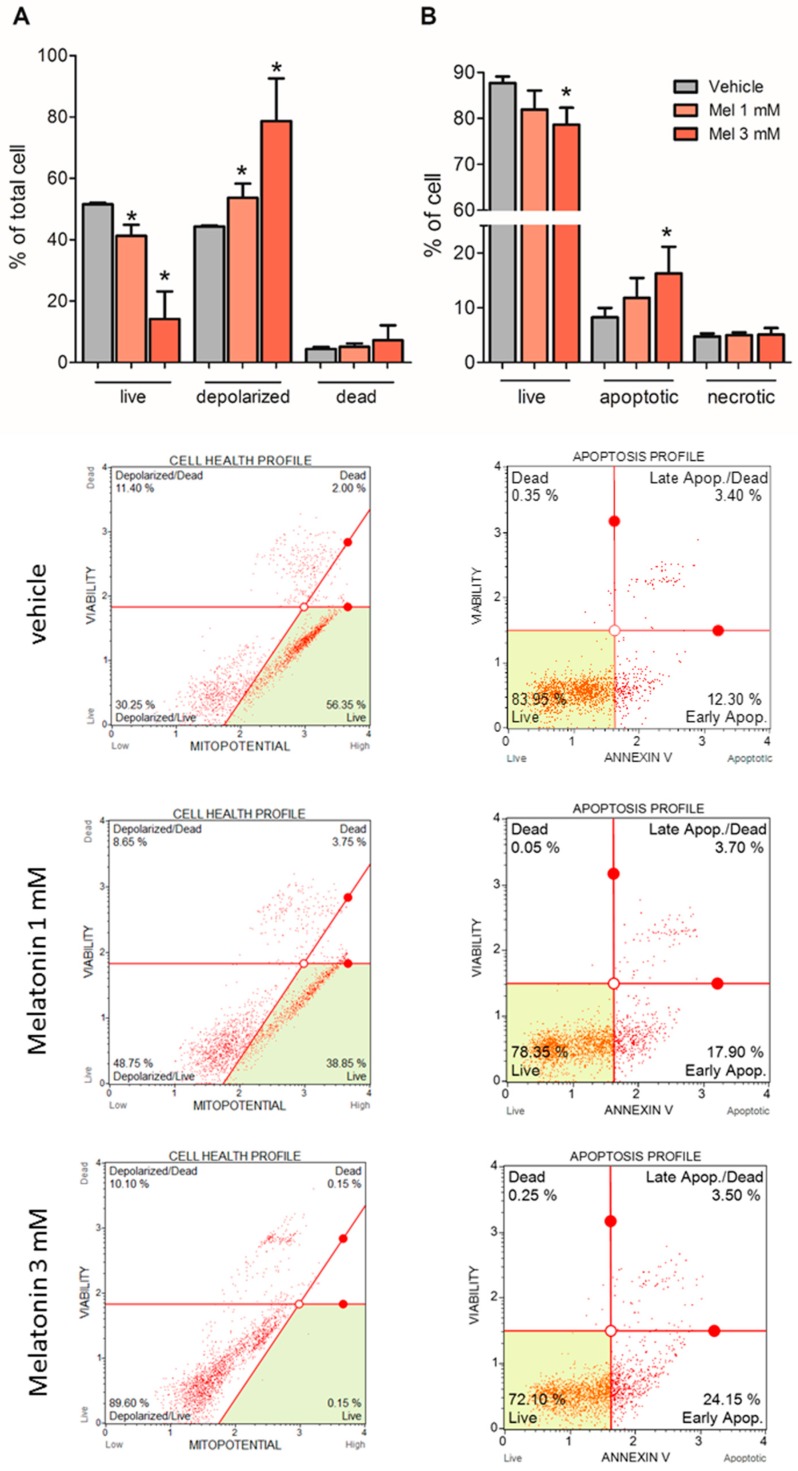
Melatonin induces mitochondrial membrane depolarization and apoptosis—U87MG cells were incubated with melatonin (1 mM or 3 mM) for 72 h, and the medium was exchanged every 24 h. (**A**) Mitochondrial polarization was evaluated by cytometry using a Muse^®^ Mitopotential Assay Kit. The depolarized group was represented by the sum of depolarized live and dead cells. Membrane polarization did not differ in cells treated with vehicle or 0.3% and 0.9% ethanol, and these groups were represented as a single bar. (**B**) Apoptosis was evaluated by cytometry using the Muse^®^ Annexin V & Dead Cell Assay Kit. The necrotic group represents the sum of dead cells and late apoptotic/dead cells presented in the representative plots. * *p* > 0.05 compared to vehicle. The statistical analysis consisted of an ANOVA followed by Bonferroni’s post-hoc test.

Two different cytometry experiments were performed to assess the ability of melatonin to induce cell death. First, the depolarization of mitochondria was measured to verify if the loss of mitochondrial inner membrane potential is associated with the early stage of apoptosis. The incubation of U87MG with 1 mM and 3 mM melatonin for 72 h decreased the percentage of live cells in the vehicle group from 51.55 ± 047% to 41.25 ± 3.61% and 14.14 ± 9.00%, respectively. Moreover, the percentage of cells with depolarized mitochondria increased from 44.25 ± 0.4% in the vehicle group to 53.65 ± 4.60% and 78.66 ± 13.87% in the melatonin-treated groups (Figure 5A). 

Second, apoptotic and necrotic cells were measured by staining phosphatidyl serine with Annexin V and 7-AAD, respectively. The results showed that the percentage of live cells in the vehicle group 86.99 ± 1.42% decreased to 83.31 ± 3.80% and 78.63 ± 3.71% in the groups treated with 1 mM and 3 mM melatonin, respectively. Specifically, the number of apoptotic cells, but not necrotic cells, increased. Apoptotic cells increased to 11.79 ± 3.61% and 16.27 ± 4.86% in the melatonin group, whereas the proportion of apoptotic cells was 8.26 ± 1.71% in the vehicle group (Figure 5B). The rates of apoptotic cells did not differ between the vehicle groups (ethanol 0.3% and 0.9%); therefore, the mean of these rates was considered as a single group. 

### 2.6. Melatonin Arrested U87MG Cells at the G0/G1 Phase of the Cell Cycle

**Figure 6:**
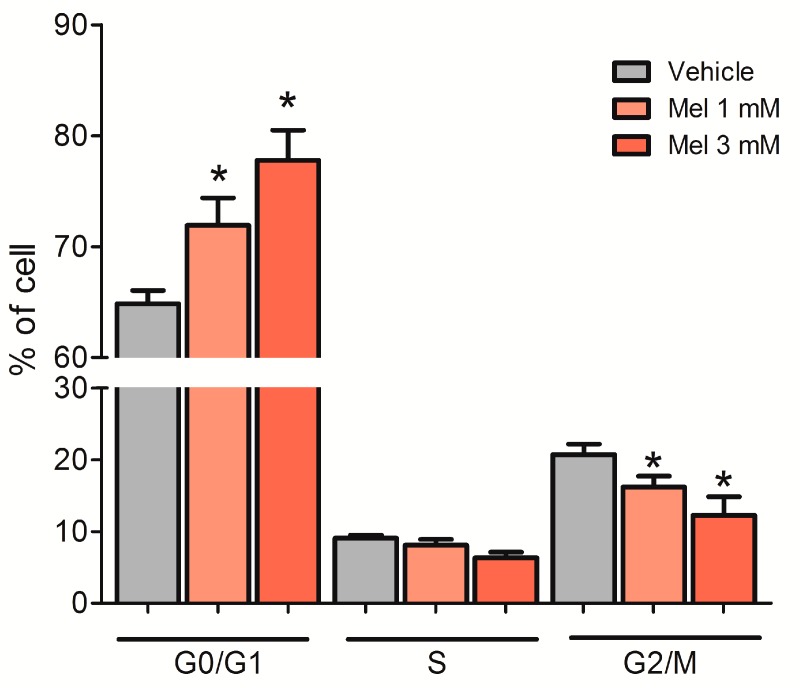
Melatonin slows the transition from the G0/G1 to S phase of the cell cycle—U87MG cells were incubated with melatonin (1 mM or 3 mM) for 72 h, and the medium was exchanged every 24 h. The cell cycle phases were evaluated by cytometry using the Muse^®^ Cell Cycle Assay Kit. The cell cycle distribution did not differ in cells treated with vehicle or 0.3% and 0.9% ethanol, and these groups were represented as a single bar. * *p* > 0.05 compared to vehicle. The statistical analysis consisted of an ANOVA followed by Bonferroni’s post-hoc test.

In addition to the effect of melatonin on apoptosis, we investigated its ability to alter the cell cycle. Accordingly, U87MG cells were incubated with either 1 mM or 3 mM melatonin for 72 h. Melatonin increased G0/G1 cell cycle arrest in U87MG cells at both 1 mM (71.92 ± 2.47%) and 3 mM (77.77 ± 2.73%), compared to the vehicle group (64.85 ± 1.20%) (Figure 6).

### 2.7. Melatonin Potentiated the Effect of Temozolomide (TMZ) to Reduce Cell Viability 

**Figure 7:**
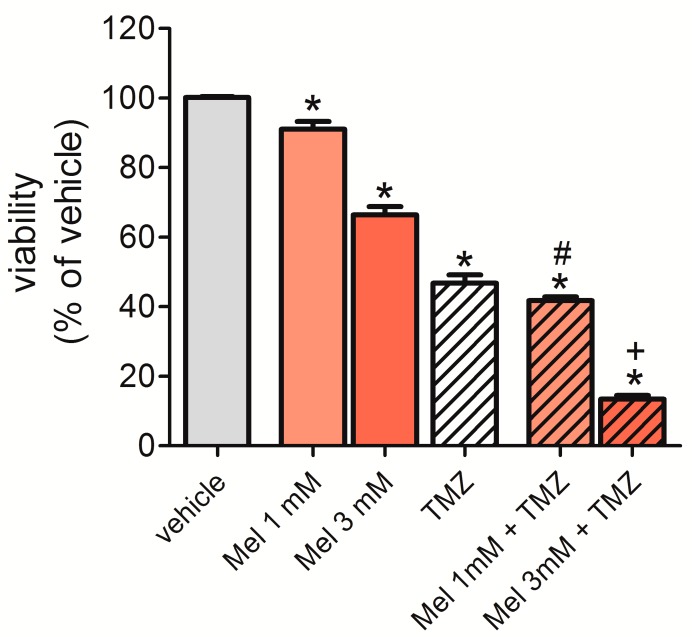
Melatonin potentiates the effect of temozolomide (TMZ) on cell proliferation/survival—U87MG cells were incubated with melatonin (1 mM or 3 mM) in combination or not with TMZ (0.8 mM) for 72 h, and the medium was exchanged every 24 h. Proliferation was assessed based on the reaction with PrestoBlue (Thermo Fisher Scientific), and the fluorescence was read on a GloMax^®^ 96 Microplate Luminometer (Promega Corporation). The results are presented as a percentage of the vehicle of each group. Proliferation did not differ between the vehicle-treated groups, and these groups were represented as a single bar. * *p* < 0.05 compared to vehicle, # *p* < 0.05 compared to the group treated with 1 mM melatonin, and + *p* < 0.05 compared to the group treated with 3 mM melatonin. The statistical analysis consisted of an ANOVA followed by Bonferroni’s post-hoc test.

To determine the potential synergistic effect of melatonin and the chemotherapeutic drug TMZ, we incubated U87MG cells for 72 h with 1 mM or 3 mM of melatonin in combination with TMZ (0.8 mM). Tumor cell viability was measured by detecting the fluorescence emitted by the reduction of the PrestoBlue^®^ reagent by living cells. The results obtained showed that 1 mM and 3 mM melatonin reduced cell viability/proliferation by 10% and 34%, respectively. TMZ reduced cell viability by 45%. Therefore, the addition of 1 mM and 3 mM melatonin increased the effect by 49% and 87%, respectively (Figure 7). Cells treated with vehicles (ethanol 0.3% or 0.9%, dimethylsulfoxide (DMSO) 0.1%, or a combination thereof) are represented as a single bar in Figure 6 because these vehicles did not affect cell viability and the viability did not significantly differ between these groups.

## 3. Discussion

Mitochondria are central organelles in the development of cancer because they are responsible for the balance of bioenergetic and biosynthetic processes, and because they are the main source of superoxides and are implicated in the intrinsic apoptosis pathway. Besides that, the transcription factor TFAM is essential for the replication, transcription, and maintenance of mtDNA and, therefore, for mitochondrial homeostasis [8,9,10]. Recent discoveries have shown that mitochondria are a target for melatonin [28], and melatonin has been shown to accumulate in mitochondria against the concentration gradient via active transport [29]. This phenomenon remains poorly understood, but melatonin is known to prevent the inhibition of complexes I and IV induced by red ruthenium [30]. In the present study, we showed that TFAM and other mitochondrial transcription factors (TFB1M and TFB2M) may be targets of melatonin in glioblastoma cells. The melatonin-induced reduction in the expression of these transcription factors was associated with reduced mitochondrial NADH dehydrogenase 1 (MT-ND1) gene expression. In accordance with our results, Prunet-Marcassus and colleagues [31] also showed that melatonin reduces the transcriptional content of mitochondria by 44% in brown adipocytes in the Siberian hamster.

Evidence indicates that TFAM has been shown to regulate mtDNA copy number [10,11], and an unbalance in the number of mtDNA copies is associated with several neurodegenerative diseases and cancer, including glioblastoma [13,32,33,34]. Melatonin did not affect the mtDNA copy number in this study, despite reducing mitochondrial gene expression, which indicates that different mechanisms are responsible for the control of mitochondrial gene expression and mtDNA replication by melatonin. In cultures of mouse C6 gliomas and Neuro2a mice, melatonin reverses the morphine- and nickel chloride-induced reductions in mtDNA copy number, respectively. But, in accordance with our results, melatonin alone does not interfere with mtDNA content [35,36].

Changes in mitochondrial gene expression or nuclear genes may result in the collapse of the mitochondrial respiratory chain to increase ROS production, which, consequently, may trigger the activation of apoptosis [3,4,5,37,38,39,40]. Our results showed that melatonin increased ROS production, which may be a consequence of alterations in the expression of respiratory chain genes that depend on the activity of the transcription factors TFAM, TFB1M, and TFB2M. This hypothesis might be supported by the fact that the downregulation of TFAM expression has promoted ROS-dependent activation of JNK/p38 MAPK and apoptosis, as reported in non-small cell lung cancer [41]. In addition, in the cardiac muscle cell line, the downregulation of TFAM caused mitochondrial oxidative phosphorylation dysfunction, resulting in increased ROS production [42]. Still, the overexpression of TFAM inhibited mitochondrial ROS generation in HeLa cells [43] and prevented oxidative stress, facilitating cardioprotection [44].

High concentrations of ROS can damage mtDNA and nuclear DNA and alter the expression of oncogenes and tumor suppressor genes, which modifies the onset and progression of tumors [37]. Several studies demonstrated that melatonin influences the intracellular content of ROS by different mechanisms. The protective role of melatonin by reducing oxidative stress in experimental models of tissue damage is well known [24]. For example, in contrast to our results, in a non-cancerous model of myocardial ischemia/reperfusion injury in type 1 diabetic rats, melatonin has increased TFAM expression, reducing mitochondrial oxidative stress and enhancing its biogenesis [45]. However, in cancer cells, two scenarios have been shown: A pro-oxidant activity in which melatonin induces the increase of intracellular levels of ROS, leading to cell death, as was shown in the present study and by others [26,46,47,48,49,50,51,52,53,54]; and melatonin reducing intracellular levels of ROS and inducing cell death by different mechanisms, such as, for example, inhibiting the nuclear transcription factor kappa B (NF-κB) nuclear activity, as reported in human glioma cells (T98 and U251) by Wang and colleagues [55], and in rat glioma cells (C6) by Martín and colleagues [56]. Otherwise, the melatonin activation of NF-κB has also been associated with an increase in intracellular oxidative stress in a model of human monocyte (U937) culture [47,48], and in a primary cerebellar granule cell culture [57]. In the present study, a known antioxidant, NAC, completely inhibited the decreased viability induced by 1 mM melatonin in a U87MG cell culture. At a melatonin concentration of 3 mM, NAC inhibited this effect by about 40%, indicating that melatonin-induced ROS increase is at least in part responsible for the observed cell death. It is important to consider the concentration and the time of exposure to melatonin. The pharmacological concentrations of melatonin used (1 mM and 3 mM) indicate that the observed effects are independent of melatonin receptors (MT1 and MT2). Minor concentrations of melatonin (1 µM and 100 µM) had no effect on cell proliferation or on the expression of TFAM (data not shown). The concentration of 3 mM of melatonin presented a more consistent result regarding the reduction of TFAM at the protein level. Although not statistically significant, a trend of TFAM protein decrease was also observed at the concentration of 1 mM (supplemental material). Interestingly, 1 mM of melatonin was enough to alter the mitochondrial gene (MT-ND1) expression. The exposition time to melatonin also proved to be crucial for the observed results. Incubation times with melatonin of less than 72 h were not sufficient to alter cell viability, as well as intracellular ROS content (data not shown). Wang and colleagues [55] have also shown no change in cell proliferation after 24 h of incubation with melatonin, although they have detected a decrease in intracellular ROS levels.

Our results showed that mitochondrial membrane depolarization was increased in cells incubated with melatonin, which indicates a collapse of inner membrane polarization that triggers the opening of mitochondrial transition pores (MTPs) and the release of cytochrome C and other pro-apoptotic factors [28,38,58,59]. The increase in ROS production induced by melatonin might lead to mitochondrial membrane depolarization and the activation of cell death, although confirmation of the activation of intrinsic apoptosis needs to be proven. Melatonin is known to affect MTP in non-cancer cells, such as in striatal neurons. In this model, melatonin prevents loss of mitochondrial membrane potential and reduces the probability of MTP opening, which prevents cell death by apoptosis [60]. Whereas in human promyelocytic leukaemia HL-60 cells, melatonin increases H_2_O_2_-induced ROS generation, causing a decrease in mitochondrial membrane potential and cell death [51]. The opposing effects of melatonin on cancer and non-cancer cells has been widely discussed and can be reviewed in [23,26].

In addition to the effects on oxidative stress and cell death, we showed that melatonin inhibits the progression of the cell cycle in U87MG cells. A possible pathway to explain this cell cycle arrest is through the physical interaction between the tumor suppressor protein p53 and TFAM [61,62,63,64]. Furthermore, p53 is a target of melatonin, which activates p53, and in its turn induces apoptosis and arrests tumor cells in the G1/G0 to S transition of the cell cycle [26,65].

Glioblastoma is a very aggressive type of cancer with a very low survival rate. Therefore, new therapeutic targets have been investigated, and TFAM is a strong candidate target since its expression is altered in several types of cancer, including glioma [13,15], colorectal cancer [63], epithelial ovarian carcinoma [66], bladder cancer [67], breast cancer [68], lung cancer [41,69], and colon cancer [70]. Moreover, the use of melatonin in the treatment of cancer has shown promising results. Specifically, previous studies have demonstrated a possible antitumor role for melatonin in glioma models [47,48]. In addition, Kinker and colleagues [71] have recently demonstrated that human glioma cell lines (HOG, T98G, and U87MG) produce melatonin, and the ability of cells to produce this hormone negatively correlated with tumor malignancy. 

Finally, our results showed that the combination of melatonin with temozolamide, TMZ, potentiated its effects on cell survival, pointing at a promising combinatorial treatment for glioblastoma patients. In summary, our results suggest that increased generation of melatonin-induced intracellular ROS in U87MG glioblastoma cells may be an effect of melatonin on the expression of TFAM and other mitochondrial transcription factors (TFB1M and TFB2M), leading to mitochondrial disruption. Our study opens a new perspective to understand the mechanism of action of melatonin in tumor cells.

## 4. Materials and Methods 

### 4.1. Cell Culture Conditions

The human malignant glioma cell line U87MG (*American Type Culture Collection, ATCC*) was routinely cultured in Dulbecco’s modified Eagle’s medium (DMEM) (Life Technologies, Carlsbad, CA, USA), supplemented with 10% fetal bovine serum (FBS) (Life Technologies), 100 IU/mL penicillin, and 100 μg/mL streptomycin (Life Technologies), in a humidified atmosphere consisting of 5% CO_2_ in air at 37 °C. The cell line was authenticated by short tandem repeat DNA profiling using the GenePrint 10 System (Promega, Madison, WI, USA).

### 4.2. Extraction of RNA and DNA

Cells were plated 2 × 10^5^ cells/mL in a 24-well plate and treated with 1 mM or 3 mM of melatonin for 72 h, and the control groups were treated with 0.3% or 0.9% of ethanol vehicle, respectively. The medium containing melatonin or vehicle was changed every 24 h. The cells were then digested with RLT Plus buffer (QIAGEN, Hilden, Germany), syringe homogenized 10 times, and frozen at −80 °C before extracting the genetic material. Both RNA and DNA were extracted from the cell homogenate using the AllPrep DNA/RNA Micro Kit (QIAGEN) following the protocol provided by the manufacturer. The concentration (ng/μL) and purity of DNA and total RNA were determined by quantification on the NanoDrop ND-1000 spectrophotometer (Thermo Scientific, Wilmington, DE, USA). 

### 4.3. Expression of TFAM, TFB1M, TFB2M, and NADH Dehydrogenase 1 (MT-ND1) by qRT-PCR

RNA was reverse transcribed using the Maxima First Strand cDNA Synthesis kit for qRT-PCR (Thermo Scientific) according to the manufacturer’s specifications. Quantitative data were obtained using SYBR green (Thermo Scientific) qRT-PCR on the ABI Prism 7500 sequence detector (Applied Biosystems, Foster City, CA, USA), and normalized in relation to the geometric mean of three housekeeping genes: Hypoxanthime phosphoribosyltransferase (HPRT), glucuronidase beta (GUSB), and TATA binding protein (TBP). The equation 2^−ΔΔCt^ was applied to calculate the relative gene expression levels [27]. The primers were designed to amplify 80–150 bp length amplicons, had a melting temperature of 60 °C, and were synthesized by Integrate DNA Technology (IDT, Coralville, IA, USA) as follows (5′ to 3′); TFAM F: CTCCCCCTTCAGTTTTGTGT, TFAM R: GCATCGGG-TTCTGAGCTTT; TFB1M F: ATGGCTCAGTACCTCTGCAATG, TFB1M R: TGGGCTGTATCAAGGGAGTGA; TFB2M F: ATCCCGGAAATCCAGACTTGT, TFB2M R: GACCAAGGCTCCATGTGCA; NADH dehydrogenase 1 (MT-ND1) F: TGATGGCTAGGGTGACTTCAT, MT-ND1 R: CCTAGCCGTTTACTCAATCCT; HPRT F: TGAGGATTTGGAAAGGGTGT, HPRT R: GAGCACACAGAGGGCTACAA; GUSB F: GAAAATACGTGGTTGGAGAGCTCATT, GUSB R: CCGAGTGAAGATCCCCTTTTTA; TBP F: AGGATAAGAGAGCCACGAACCA, TBP R: CTTGCTGCCAGTCTGGACTGT. PCR was carried out as follows: 5 min at 50 °C, 10 min at 95 °C, 40 cycles at 95 °C for 15 s, and 60 °C for 1 min. The primer concentrations used were 200–400 nM. All assays were carried out in duplicate and eventually repeated when the standard deviation exceeded 0.4. 

### 4.4. Mitochondrial DNA Copy Number Quantification

A single copy gene—hemoglobin beta (HBB)—was used as a reference to determine the number of copies of mtDNA by SYBR Green qRT-PCR on an ABI Prism 7500 sequence detector (Applied Biosystems). The primer sequence used to quantify the mtDNA copy number was the same as that of NADH dehydrogenase 1, and the primer was used at a final concentration of 200 nM. The sequences of HBB were as follows (5′–3′): HBB F: GTGAAGGCTCATGGCAAGA and HBB R: AGCTCACTCAGGTGTGGCAAAG (IDT). The cycle conditions were 10 min at 95 °C, 40 cycles at 95 °C for 15 s, and 60 °C for 1 min. All assays were carried out in duplicate and eventually repeated when the standard deviation exceeded 0.4. The relative mtDNA copy number was determined with the equation 2^−ΔΔCt^ [27].

### 4.5. Western Blot Analysis 

Total protein lysates were prepared from U98MG cell cultures with RIPA lysis buffer and protease inhibitor cocktail (Sigma-Aldrich) on ice. The protein concentration was determined using a NanoDrop Microvolume Spectrophotometers (Thermo Scientific™). Total protein lysates (30 mg) were separated by 4% to 12% polyacrylamide gel electrophoresis (Invitrogen, Carsbald, CA, USA) with 1× NuPAGE MOPS SDS 20× (Invitrogen, Carsbald, CA, USA) running buffer. The proteins were electrophoretically transferred to a Polyvinylidene Fluoride membrane (PVDF) through the semi-dry Trans-Blot^®^ SD system (Trans-Blot^®^ Transfer Cell, Biorad, Hercules, CA, USA). The membrane was blocked with 5% skim milk and incubated with rabbit monoclonal primary anti-TFAM diluted 1:500, and with mouse monoclonal primary anti-β-actina (clone AC-74, Sigma-Aldrich) diluted 1:5000, as a protein loading control. The secondary antibodies used were anti-rabbit (1:1000) and anti-mouse IgG (1:5000) conjugated to peroxidase (Sigma-Aldrich). The immune complexes were visualized using enhanced chemiluminescence reagent (Western Lightning Chemiluminescence Reagent Plus, Perkin Elmer, Waltham, MA, USA) and detected with UVITEC (Alliance 4.7) Cambridge, UK.

### 4.6. Evaluation of Oxidative Stress, Cell Cycle, Apoptosis, and Mitochondria Polarization

U87MG cells were treated with 1 mM or 3 mM melatonin for 72 h. Control groups were treated with ethanol vehicle (0.3% or 0.9%, relative to the melatonin concentration). The medium was changed every 24 h, and the melatonin or vehicle was replaced. The cells were then prepared for cytometric assays to evaluate oxidative stress, the cell cycle, apoptosis, and mitochondrial membrane polarization using the Muse^®^ Cell Analyzer (Merck Millipore, Billerica, MA, USA) and appropriate reagent kits: Muse^®^Cell Oxidative Stress Kit, Muse^®^Cell Cycle Assay Kit, Muse^®^Annexin V & Dead Cell Assay Kit and Muse^®^ Mitopotential Assay Kit, respectively, according to the manufacturer’s instructions.

### 4.7. Cell Viability/Proliferation

U87MG cells were treated with melatonin (1 mM or 3 mM) in the presence or absence of temozolomide (TMZ 0.8 mM—Sigma-Aldrich, St. Louis, MO, USA) or *N*-Acetyl-l-cysteine (NAC 10 mM—Sigma-Aldrich, St. Louis, MO, USA) for 72 h, and the medium and drug were replaced every 24 h. The control groups were treated with melatonin vehicle (0.3% or 0.9% ethanol), TMZ vehicle (0.1% dimethylsulfoxide—DMSO), or a combination thereof (0.3% ethanol + DMSO 0.1% or 0.9% ethanol + 0.1% DMSO). NAC was diluted in DMEM. After the treatments, the cells were incubated with PrestoBlue reagent (Invitrogen, Carlsbad, CA, USA) for 2 h, and the fluorescence was measured on a GloMax^®^ 96 Microplate Luminometer (Promega Corporation, Madison, WI, USA).

### 4.8. Statistical Analysis

The results are reported as the mean ± s.e.m. of at least three independent experiments, and were normalized to the groups treated with specific vehicles for each experiment. The differences between experimental groups were tested with an analysis of variance followed by the Bonferroni post-hoc correction using GraphPad Prism^®^ version 5.

## Supplementary Materials

The following are available online.
