# Supplementary material for: Mitochondria Transcription Factor A: A Putative Target for the Effect of Melatonin on U87MG Malignant Glioma Cell Line
Source: Molecules. 2018 May 9;23(5):1129. doi: 10.3390/molecules23051129 (PMC6099566; doi:10.3390/molecules23051129)
Supplement: Supplementary file 1 [file molecules-23-01129-s001.pdf]

Table 1. From left to right, relative expression of TFAM, TFB1M and TFB2M after vehicle (0.3 % or 0.9 %) or melatonin (1mM or 3 mM) treatment for 72 hours. Data are expressed as the relative quantification ($2^{-\Delta\Delta Ct}$) compared to the respective vehicle-treated groups.

	TFAM		
	vehicle	Mel 1 mM	Mel 3 mM
	1,00	0,87	0,70
	1,00	1,02	0,37
	1,00	0,83	0,85
	1,28	0,82	0,64
	0,78	0,88	0,58
	1,29	0,27	0,82
	0,78	0,24	
	1,00	0,58	
	0,98	1,05	
	1,02		
mean	1,01	0,73	0,66
s.e.m.	0,05	0,10	0,07

	TFB1M		
	vehicle	Mel 1 mM	Mel 3 mM
	1,00	0,53	0,35
	0,90	0,35	0,34
	1,11	0,49	0,55
	1,19		
	0,84		
	0,94		
	1,07		
	0,76		
	1,32		
	1,26		
mean	1,04	0,46	0,41
s.e.m.	0,06	0,05	0,07

	TFB2M		
	vehicle	Mel 1 mM	Mel 3 mM
	1,00	0,55	0,39
	0,81	0,47	0,35
	1,24	0,47	0,68
	1,35		
	0,74		
	0,99		
	1,01		
	1,08		
	0,92		
	1,01		
mean	1,02	0,50	0,47
s.e.m.	0,05	0,03	0,10

Fig 1. On the top, representative Western blot images showing the effects of melatonin treatment (1 mM or 3 mM) and their respective vehicle groups (etanol 0.3 % or 0.9 %) on the protein TFAM expression. Four independent experiment are identified by colors. Table 2. At bottom, percentage of quantitative signal intensities of the protein TFAM expression after normalization with β-actina.

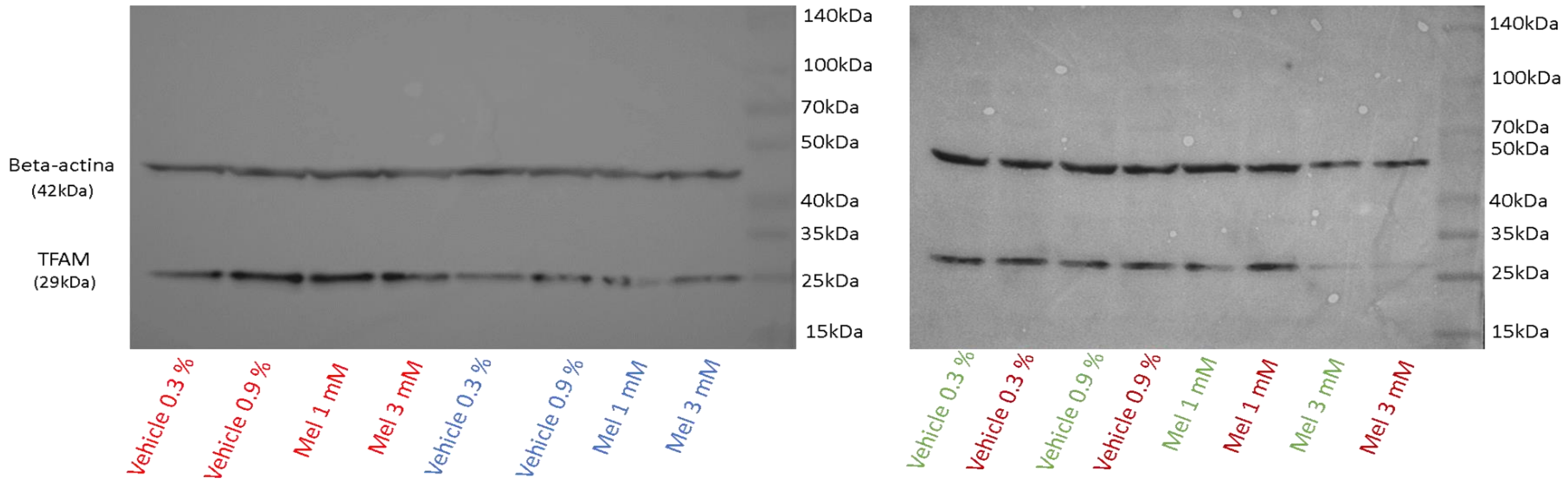

number of experiment	vehicle 0.3 %	vehicle 0.9 %	Mel 1 mM	Mel 3 mM
1	100,00	100,00	106,47	64,79
2	100,00	100,00	72,38	80,32
3	100,00	100,00	65,22	46,39
4	100,00	100,00	103,65	31,11
média	100,00	100,00	86,93	55,65
s.e.m.	0,85	1,83	10,58	10,72

Table 3. From left to right, relative expression of the NADH dehydrogenase 1 gene (MT-ND1) and mtDNA copy number. Data are expressed as the relative quantification ($2^{-\Delta\Delta Ct}$) compared to the respective vehicle-treated groups (ethanol 0.3 % or 0.9 %)

	MT-ND1		
	vehicle	Mel 1 mM	Mel 3 mM
	0,97	0,44	0,81
	1,03	0,55	0,42
	0,99	0,64	0,69
	1,01		
	0,81		
	1,23		
mean	1,01	0,54	0,64
s.e.m.	0,05	0,06	0,12

	mtDNA		
	vehicle	Mel 1 mM	Mel 3 mM
	0,93	1,02	0,83
	0,92	1,17	1,10
	1,16	0,90	1,50
	0,88	1,17	1,21
	1,13	0,71	1,51
	0,86	1,38	0,99
	1,16	1,24	0,99
	1,17		0,40
	0,85		0,51
	1,11		
	0,90		
	0,86		
	1,16		
	0,97		
	1,03		
	1,04		
	0,96		
	1,01		
	0,99		
mean	1,00	1,08	1,00
s.e.m.	0,03	0,09	0,13

Table 4. From left to right, ROS production was assessed by cytometry using the Muse®Cell Oxidative Stress kit. The cells U87MG were treated with vehicle (0.3% or 0.9 % ethanol) or melatonin (1 mM or 3 mM) for 72 hours. The results are presented as the percentage of cells positively labeled for superoxide radicals. And cell proliferation that was assessed based on the reaction with PrestoBlue (Thermo Fisher Scientific), and the fluorescence was read on a GloMax® 96 Microplate Luminometer (Promega Corporation). The results are presented as a percentage of the control group - sum of vehicle 0.3 and 0.9 % of ethanol and NAC vehicle (water) - as the proliferation did not differ between the vehicle-treated groups.

	% of cells ROS positive		
	vehicle	Mel 1 mM	Mel 3 mM
	18,44	19,24	32,43
	18,27	19,03	17,18
	8,34	19,52	21,25
	10,47	25,73	
	19,23	20,77	
	15,07	20,07	
mean	14,97	20,73	23,62
s.e.m.	1,87	1,03	4,56

	proliferation/survival					
	control	Mel 1 mM	Mel 3 mM	NAC	Mel 1 mM + NAC	Mel 3 mM + NAC
	120	79	32	94	94	67
	105	79	35	91	103	71
	98	73	36	93	101	72
	94	73	28	85	92	68
	96					
	100					
	93					
	93					
mean	100,0	76,0	32,8	90,8	97,5	69,5
s.e.m.	3,2	1,7	1,8	2,0	2,7	1,2

Table 5. Mitochondrial polarization and apoptosis were evaluated by cytometry using the Muse® Mitopotential Assay Kit and the Muse® Annexin V & Dead Cell Assay Kit, respectively. The cells U87MG were treated with respecitve vehicle (0.3% or 0.9 % ethanol) or melatonin (1 mM or 3 mM) for 72 hours.

Mitochondrial membrane depolarization

	% of live cells			% of depolarized cells			% of dead cells		
	vehicle	Mel 1 mM	Mel 3 mM	vehicle	Mel 1 mM	Mel 3 mM	vehicle	Mel 1 mM	Mel 3 mM
	50,62	48,35	30,93	43,71	44,50	52,48	5,67	7,15	16,59
	52,13	36,55	11,35	44,05	59,05	83,80	3,83	4,40	4,85
	51,90	38,85	0,15	45,00	57,40	99,70	3,70	3,75	0,15
mean	51,55	41,25	14,14	44,25	53,65	78,66	4,40	5,10	7,20
s.e.m.	0,47	3,61	9,00	0,39	4,60	13,87	0,64	1,04	4,89

Apoptosis

	% of live cells			% of apoptotic cells			% of necrotic cells		
	Veic	Mel 1 mM	Mel 3 mM	Veic	Mel 1 mM	Mel 3 mM	Veic	Mel 1 mM	Mel 3 mM
	84,77	87,47	89,00	9,17	7,00	2,30	6,07	5,53	8,70
	89,20	89,50	72,10	8,15	6,65	24,15	2,65	3,85	3,75
	86,50	87,85	74,80	10,25	8,10	21,30	3,25	4,05	3,90
	92,70	91,80	78,60	1,25	1,75	17,33	6,05	6,45	4,06
	92,45	78,35		0,90	17,90		6,65	3,75	
	82,05	62,45		14,35	30,40		3,60	7,15	
	83,95	85,23		12,30	10,73		3,75	4,05	
	84,32			9,70			5,99		
mean	86,99	83,24	78,63	8,26	11,79	16,27	4,75	4,98	5,10
s.e.m.	1,42	3,80	3,71	1,71	3,61	4,86	0,56	0,53	1,20

Table 6. Cell cycle phases were evaluated by cytometry using the Muse® Cell Cycle Assay Kit. The cells U87MG were treated with respective vehicle (0.3% or 0.9 % ethanol) or melatonin (1 mM or 3 mM) for 72 hours.

	Cell Cycle								
	% of cells in G0/G1 phase			% of cells in S phase			% of cells in G2/M phase		
	vehicle	Mel 1 mM	Mel 3 mM	vehicle	Mel 1 mM	Mel 3 mM	vehicle	Mel 1 mM	Mel 3 mM
	63,50	69,40	72,30	8,60	11,00	7,90	24,70	17,80	17,50
	65,90	78,60	80,40	7,80	6,00	5,70	18,20	13,30	9,70
	62,30	79,70	80,60	7,20	5,90	5,40	17,20	12,00	9,50
	61,30	69,80		9,60	7,60		17,10	19,90	
	68,50	70,00		9,60	8,60		25,80	18,00	
	67,60	64,00		9,90	9,50		26,10		
				10,60			19,00		
							17,80		
mean	64,85	71,92	77,77	9,04	8,10	6,33	20,74	16,20	12,23
s.e.m.	1,20	2,47	2,73	0,46	0,82	0,79	1,43	1,51	2,63

Table 7. Cell proliferation/survival was assessed based on the reaction with PrestoBlue (Thermo Fisher Scientific), and the fluorescence was read on a GloMax® 96 Microplate Luminometer (Promega Corporation). The results are presented as a percentage of the vehicle of each group (vehicle of melatonin 1 mM and 3 mM = 0.3 or 0.9 % of ethanol, respecively; vehicle of TMZ 0.8 mM = 0.1 % of DMSO)

	proliferation/survival					
	vehicle	Mel 1 mM	Mel 3 mM	TMZ	Mel 1mM + TMZ	Mel 3 mM + TMZ
	100,00	94,16	68,08	61,70	48,69	17,75
	101,00	90,07	70,78	45,68	41,48	14,03
	99,00	95,53	70,22	46,38	39,64	14,12
	100,00	96,57	66,81	47,64	39,60	13,63
	101,00	95,66	70,47	45,99	39,75	15,18
	99,00	93,21	68,80	40,82	39,31	13,23
	100,00	80,78	65,64	40,10	43,26	12,27
	101,00	82,26	50,25	46,08	42,11	7,50
mean	100,13	91,03	66,38	46,80	41,73	13,46
s.e.m.	0,30	2,20	2,39	2,34	1,12	1,03
